# Amphetamines Improve the Motivation to Invest Effort in Attention-Deficit/Hyperactivity Disorder

**DOI:** 10.1523/JNEUROSCI.0982-23.2023

**Published:** 2023-10-11

**Authors:** Trevor T.-J. Chong, Erika Fortunato, Mark A. Bellgrove

**Affiliations:** ^1^Turner Institute for Brain and Mental Health, School of Psychological Sciences, Monash University, Clayton, Victoria 3800, Australia; ^2^Department of Neurology, Alfred Health, Melbourne, Victoria 3004, Australia; ^3^Department of Clinical Neurosciences, St Vincent’s Hospital, Melbourne, Victoria 3065, Australia

**Keywords:** ADHD, amphetamines, dopamine, effort, motivation, noradrenaline

## Abstract

Prevailing frameworks propose that a key feature of attention-deficit/hyperactivity disorder (ADHD) is lower motivation. An important component of motivation is the willingness to engage in cognitively or physically effortful behavior. However, the degree to which effort sensitivity is impaired in ADHD has rarely been tested, and the efficacy of stimulant medication in ameliorating any such impairments is unclear. Here, we tested 20 individuals with ADHD (11 males, 9 females) who were managed with amphetamine-based medication (dexamfetamine, lisdexamfetamine), and 24 controls (8 males, 16 females). Individuals with ADHD were tested over two counterbalanced sessions, ON and OFF their usual amphetamine-based medication. In each session, participants performed an effort-based decision-making task, in which they were required to choose how much cognitive or physical effort they were willing to engage in return for reward. Our results revealed three main findings. First, individuals with ADHD had lower motivation relative to controls to invest effort in both the cognitive and physical domains. Second, amphetamine increased motivation uniformly across both domains. Finally, the net effect of amphetamine treatment was to mostly restore motivation across both domains of effort relative to healthy controls. These data provide clear evidence for a heightened sensitivity to both cognitive and physical effort in ADHD, and reveal the efficacy of amphetamine-based drugs in restoring effort sensitivity to levels similar to controls. These findings confirm the existence of reduced motivational drive in ADHD, and more broadly provide direct causal evidence for a domain-general role of catecholamines in motivating effortful behavior.

**SIGNIFICANCE STATEMENT** A core feature of attention-deficit/hyperactivity disorder (ADHD) is thought to be a heightened aversion to effort. Surprisingly, however, the degree to which effort sensitivity is impaired in ADHD has rarely been tested. More broadly, the relative efficacy of catecholamines in motivating the investment of cognitive and physical effort is unclear. We tested 20 individuals with ADHD ON and OFF amphetamines, and compared their behavior on an effort-based decision-making task to 24 controls. When tested OFF medication, the ADHD group was less cognitively and physically motivated than controls. However, amphetamines led to a comparable increase in motivation across both domains. This demonstrates the efficacy of catecholamines in facilitating domain-general effort, and highlights the broader potential of such drugs to treat disorders of motivation.

## Introduction

Attention-deficit/hyperactivity disorder (ADHD) is a disorder of catecholamine dysregulation that affects multiple aspects of cognition (Faraone and Biederman, 1998; Hervey et al., 2004; Boonstra et al., 2005; Faraone et al., 2015). Contemporary frameworks propose that some of the key cognitive impairments in ADHD are driven by reduced motivation. In particular, it has long been suggested that the motivational changes in ADHD are characterized by an aversion toward effortful behavior (Sonuga-Barke, 2003; Sergeant, 2005; American Psychiatric Association, 2022). Effort can be experienced across multiple domains, for example, it can be perceived both cognitively (e.g., when studying for an examination) and physically (e.g., when training for a race). However, despite the importance of impaired motivation in prevailing frameworks of ADHD, there has been limited data to suggest that effort allocation is reduced, either cognitively or physically.

A common approach to studying motivation in ADHD is with subjective self-reports, which are limited in their capacity to provide a mechanistic account (Carlson and Tamm, 2000; Volkow et al., 2011). Experimental measures of motivation have primarily examined the sensitivity of individuals to reward (Nigg, 2005; Tripp and Wickens, 2009; Luman et al., 2010), typically with delay discounting paradigms that show individuals with ADHD have a greater preference for rewards that are smaller, but more immediate, than those that are larger, but more delayed (Sonuga-Barke et al., 2008; Jackson and MacKillop, 2016; Patros et al., 2016). In the broader literature on goal-directed behavior, however, reward sensitivity is only one of several distinct components of motivation (Husain and Roiser, 2018; Chong, 2020). A sensitive and complementary measure is the willingness to engage in effortful behavior, a measure that is behaviorally and neurally dissociable from reward sensitivity (Le Bouc et al., 2016; Meyniel et al., 2016; Chong et al., 2018b).

The idea that impaired effort allocation is a key feature of ADHD was first advanced nearly 20 years ago (Sergeant, 2005). In that time, however, this proposal has rarely been empirically tested. In particular, no study in ADHD has systematically examined the aversiveness of behavior that is cognitively effortful. This is a critical omission, given that current diagnostic criteria for ADHD emphasize that a key characteristic is precisely the avoidance, dislike or reluctance to engage in mentally effortful tasks (American Psychiatric Association, 2022). The only studies that have examined effort aversion in ADHD have been in the context of physical effort. Even so, only three studies have been reported, of which two found no differences in effort sensitivity between ADHD and controls (Winter et al., 2019; Mies et al., 2018), and one applied a task that was unable to distinguish effort from delay discounting (Addicott et al., 2019). In sum, extant data provide limited support for currently influential frameworks of ADHD, which assert that a lower willingness to invest effort is an important component of the disorder.

In the broader neuroscience literature, dopamine and noradrenaline are known to play causal roles in motivated behavior. Lower levels of these catecholamines are associated with lower motivation to invest effort (Salamone et al., 2016; Borderies et al., 2020), and exogenous catecholamines facilitate both cognitive (Cocker et al., 2012; McGuigan et al., 2019; Westbrook et al., 2020; Bogdanov et al., 2022) and physical effort (Wardle et al., 2011; Chong et al., 2015; Yohn et al., 2016; Addicott et al., 2019; Soutschek et al., 2020; Soder et al., 2021). However, the mechanisms underlying cognitive and physical motivation are also partially dissociable, being subserved by a network of domain-general and domain-specific mechanisms (Schmidt et al., 2012; Hosking et al., 2015; Chong et al., 2017; Atkins et al., 2020). Given that catecholaminergic dysregulation is an hypothesised core pathophysiological mechanism of ADHD (Faraone and Biederman, 1998; Faraone et al., 2015), these data predict that effort sensitivity should also be reduced in ADHD. However, it remains to be determined whether such changes are unique to single domains of effort, or generalize across them. Similarly, the relative efficacy of catecholaminergic stimulants (such as amphetamines) in restoring such deficits across different domains of effort is unknown.

## Materials and Methods

In this study, we tested whether individuals with and without ADHD differ in their motivation to exert cognitive and physical effort and, if so, the degree to which amphetamines are capable of ameliorating such differences. We recruited individuals with ADHD being managed with amphetamine-based drugs (either dexamfetamine or its prodrug, lisdexamfetamine), and tested them over two counterbalanced sessions, once on their usual treatment, and the other following a 72-h washout. Our paradigm involved training participants on cognitively and physically effortful tasks, before asking them to decide how much effort they were willing to invest in each domain for particular rewards. This allowed us to compare cognitive and physical effort-based decisions across the ADHD and non-ADHD groups, and to determine the efficacy of amphetamines in ameliorating any such differences.

### Participants

We recruited 20 individuals with ADHD (11 males, 9 females) and 24 individuals without ADHD (i.e., controls; 8 males, 16 females). ADHD participants were recruited through practicing clinicians, local community support groups, and social media. Our inclusion criteria for ADHD participants included: (1) a diagnosis of ADHD confirmed and managed by a psychiatrist; (2) a score >65 on any of Section E, F, or G on the Conners’ Adult ADHD Rating Scale (CAARS; Conners et al., 1999); (3) a score >36 on the Wender Utah Rating Scale (WURS; Ward, 1993); and (4) being managed on either lisdexamfetamine or dexamfetamine. Control participants were excluded if their scores on the CAARS and WURS fell within the above ranges. Exclusion criteria for both groups included a history of neurologic disease (including acquired brain injury), major psychiatric illness, and/or being on other psychotropic medications (e.g., anti-depressants).

Of the 20 participants with ADHD, eight were taking dexamfetamine (mean dose 35.6 mg ± SD 14.3 mg), and 12 lisdexamfetamine (55.4 ± 19.7 mg). To determine the effect of stimulant medication on behavior, individuals with ADHD were tested in two counterbalanced sessions. In one session, they were tested ON their usual medication (i.e., had taken their usual dose of stimulant medication for at least 14 d prior). In a separate session, they were tested after at least 72 h OFF medication to allow for washout (Adler et al., 2017; Boellner et al., 2010). Drug order was counterbalanced across participants, and the two sessions were separated by two to three weeks (mean 16.8 d ± SD 5.6 d). All participants were reimbursed a fixed amount for their participation, and the study was approved by the Monash University Human Research Ethics Committee.

In addition to the CAARS and WURS, we assessed trait measures of apathy and depression with the Dimensional Apathy Scale (Radakovic and Abrahams, 2014), and Hospital Anxiety and Depression Scale (Zigmond and Snaith, 1983), respectively. We tested for alcohol and drug-related problems with the Alcohol Use Disorders Test (Saunders et al., 1993), and the Drug Use Disorders Identification Test (Berman et al., 2007). We obtained global measures of intelligence and cognitive function with the National Adult Reading Test (Blair and Spreen, 1989) and Montreal Cognitive Assessment (Nasreddine et al., 2005). These data are summarized in Table 1.

**Table 1.** Summary of participant demographics [means (SD)]

	Controls	ADHD	Group difference
*N*	24	20	N/A
Age (years)	29.9 (9.80)	35.8 (11.1)	n.s.
Gender (M:F)	8:16	11:9	n.s.
Conners’ Adult ADHD Rating Scale (CAARS)			
A. Inattention/Memory	47.5 (7.41)	75.4 (9.59)	*p* < 0.001
B. Hyperactivity/Restlessness	44.0 (5.91)	63.3 (10.6)	*p* < 0.001
C. Impulsivity/Restlessness	45.8 (8.44)	65.3 (14.0)	*p* < 0.001
D. Self-concept	50.7 (11.6)	62.7 (9.43)	*p* < 0.001
E. DSM-IV Inattentive	48.8 (9.45)	81.3 (9.06)	*p* < 0.001
F. DSM-IV Hyperactive-impulsive	43.8 (7.97)	68.0 (15.3)	*p* < 0.001
G. DSM-IV ADHD Symptoms Total	46.3 (9.36)	77.2 (12.5)	*p* < 0.001
H. ADHD Index	46.8 (9.31)	69.5 (9.64)	*p* < 0.001
Wender Utah Rating Scale (WURS)	28.5 (18.8)	59.9 (16.2)	*p* < 0.001
Hospital Anxiety and Depression Scale			
- Anxiety	6.75 (3.52)	11.1 (3.15)	*p* < 0.001
- Depression	3.04 (2.09)	7.45 (3.63)	*p* < 0.001
Dimensional Apathy Scale			
- Executive apathy	8.42 (3.55)	16.3 (4.14)	*p* < 0.001
- Emotional apathy	7.17 (3.96)	9.7 (5.17)	n.s.
- Behavioral apathy	8.5 (3.88)	10.4 (3.90)	n.s.
National Adult Reading Test	20.1 (5.60)	16.8 (5.13)	*p* = 0.048
Montreal Cognitive Assessment	27.2 (1.77)	26.8 (2.02)	n.s.
Alcohol Use Disorders Test	3.33 (4.31)	4.95 (4.42)	n.s.
Drug Use Disorders Identification Test	3.25 (0.85)	4.75 (2.45)	*p* < 0.01

N/A, not applicable; n.s., not significant.

### Experimental design

**Figure 1.**
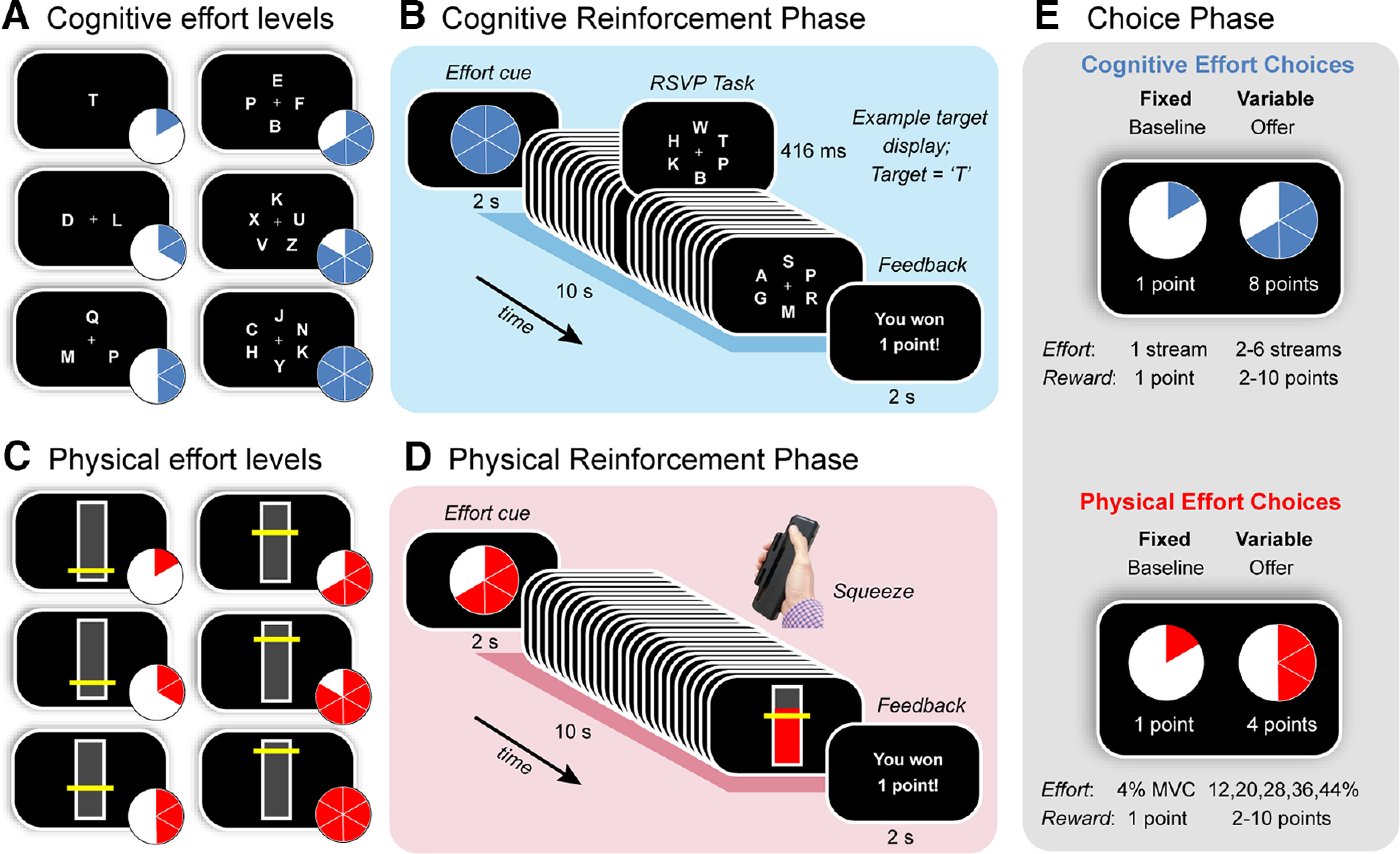
Task design. Participants were first trained on (***A***, ***B***) a cognitively effortful task, and (***C–D***) a physically effortful task, before (***E***) indicating their preference for investing effort for reward. ***A***, The cognitive effort task required participants to monitor one to six RSVP streams for a target letter (T). ***B***, Each trial began with a blue pie chart indicating the number of streams they had to monitor on that trial. After completing each effort level, participants received feedback on their performance. ***C***, The physical effort task required participants to sustain variable amounts of force on a hand-held dynamometer, with the target levels of force defined as a function of each individual’s maximum voluntary contraction (MVC; 4%, 12%, 20%, 28%, 36%, 44%). ***D***, Each trial began with a red pie chart indicating the amount of force they had to apply on that trial. Trial durations were identical to those for the cognitive effort task (10 s). At the conclusion of each trial, participants received feedback on their performance. ***E***, The choice phase required participants to decide how much effort they were willing to invest for reward. The choice was always between a fixed baseline option [the lowest level of effort for the lowest reward (1 point)], and a variable high-effort/high-reward offer (higher levels of effort; rewards of 2 to 10 points). Separate choices were made for cognitive and physical effort.

This task utilized a paradigm that has been previously applied in healthy adults (Chong et al., 2017) and clinical groups (Atkins et al., 2022). It was divided into three phases (Fig. 1). The first two (“Reinforcement”) involved training participants on separate tasks that were cognitively (Fig. 1*A*,*B*) or physically effortful (Fig. 1*C*,*D*). In each task, we varied demands in the target domain (e.g., cognitive), while keeping those in the other (e.g., physical) constant. Task order was counterbalanced. The Reinforcement phases were followed by a “Choice” phase, during which participants were asked to choose between a fixed low-effort/low-reward option, and a variable high-effort/high-reward offer (Fig. 1*E*). This allowed us to quantify the willingness of individuals to exert distinct types of effort.

#### Reinforcement phases

##### Cognitive effort task

The cognitive effort task was based on a rapid serial visual presentation (RSVP) paradigm (McGuigan et al., 2019; Atkins et al., 2022). Participants were required to monitor a series of rapidly changing letters (Arial, 26-point font, Fig. 1*A*,*B*) and press a button whenever they detected the target letter, T. We parametrically manipulated cognitive demand by increasing the number of letter streams from one to six. In the least effortful condition (level 1), a single stream was presented centrally. In the more effortful conditions (levels 2–6), between two to six streams were positioned equidistantly from fixation. The target letter could appear randomly in any stream, and the timing of the target stimuli was pseudorandom such that they could not appear in consecutive stimulus frames (to avoid an attentional blink). Each effort level comprised 24 stimulus frames, each of which lasted 416 ms, for a total trial duration of 10 s.

Each trial of the Reinforcement phase commenced with a blue pie chart, which cued the level of cognitive effort required on that trial. After completing the required level of effort, participants received feedback about their success. They were rewarded with one point if they were able to complete each trial above a threshold level of performance (more than one hit; fewer than three false alarms); otherwise, they were not rewarded. Participants were instructed that their task was to maximize the number of points won. They completed two blocks of 30 trials (10 trials per effort level, randomly allocated), with a brief rest period after each block. These experimental blocks were preceded by a practice block of 12 trials (two per effort level). Responses were registered on a Cedrus button box, and the task was implemented on Presentation software (Neurobehavioral Systems).

##### Physical effort task

In the physical effort task, participants exerted one of six levels of force on a hand-held dynamometer (SS25LA, BIOPAC Systems) using their dominant hand (Fig. 1*C*,*D*; Chong et al., 2018a; Atkins et al., 2020). At the beginning of the experiment, we determined individuals’ maximum voluntary contraction (MVC), which was defined as the maximum of three consecutive ballistic squeezes. To standardize effort requirements across participants, we defined the target effort levels for each individual as a function of their MVC (4–44%, in increments of 8%). Target levels were depicted as a horizontal yellow line on a vertical bar, and participants received real-time visual feedback of their applied force.

Each trial in the physical effort task commenced with a red pie chart, which cued the level of physical effort required on that trial. Participants then had to initiate and maintain their contraction above the required effort level for ≥50% of the total trial duration to be successfully rewarded. The physical effort task was identical to the cognitive effort task with respect to trial durations (10 s per effort level); number of trials per effort level; and overall block structure. The physical effort task was implemented on Psychtoolbox (http://psychtoolbox.org) in MATLAB (The MathWorks).

#### Choice phase

Finally, participants undertook the Choice phase, which allowed us to measure the key outcome of interest, the willingness to exert cognitive and physical effort. In this phase, participants revealed their preference between a fixed, low-effort/low-reward baseline option, and a variable, high-effort/high-reward offer. The fixed baseline option was always the option to exert the lowest amount of effort for the lowest reward (one point). In contrast, the variable offer was the option to exert a higher amount of effort (levels 2–6) for a greater reward (2–10 points). In order to separate individuals’ cognitive and physical motivation, each choice was always made between two options in the same domain. We sampled the entire effort-reward space evenly and randomly across both domains over a total of 150 trials. Participants made their selection with a button press, and trials were self-paced. To reduce the impact of fatigue on subsequent decision-making, participants were not required to execute their choices, but simply indicate their preferred option. They were explicitly told that their decisions were hypothetical, in that points did not alter remuneration, but that they should select the option that was most preferable to them. This protocol is consistent with previous studies (Skvortsova et al., 2017; McGuigan et al., 2019; Atkins et al., 2020).

### Statistical analysis

In the reinforcement phase, we compared differences in performance between groups (ADHD vs controls) or drug (ADHD ON vs OFF) with separate repeated-measures ANOVAs. Specifically, when comparing group differences, we analyzed performance with a Group (either ADHD ON vs controls, or ADHD OFF vs controls) × Effort (levels 1–6) ANOVA. We compared the effect of Drug with an analogous Drug (ON vs OFF) × Effort ANOVA. Violations to the assumption of sphericity were managed with Greenhouse–Geisser corrections, and *post hoc* pairwise comparisons were corrected for multiple comparisons with the Bonferroni approach.

In the choice phase, our core approach to analyze the choice data were with a generalized linear mixed effects model, which allowed us to assess all variables of interest on every trial. We used a logistic transform to analyze Choice (baseline, offer) as the outcome variable. Across all analyses, we entered Domain (cognitive, physical), Effort (levels 2–6), and Reward (levels 2–6) as fixed effects. In separate analyses, we included either Group (ADHD OFF vs controls; ADHD ON vs controls) or Drug (ON vs OFF) as an additional fixed effect to compare the effect of each on choice. Predictor variables were Z-scored, and we modeled the full range of fixed effects and interactions. Individual subjects were fit with random intercepts. Data were analyzed using the *fitglme* function in MATLAB with a logistic link function and Laplace fit method.

## Results

### Reinforcement phase

**Figure 2.**
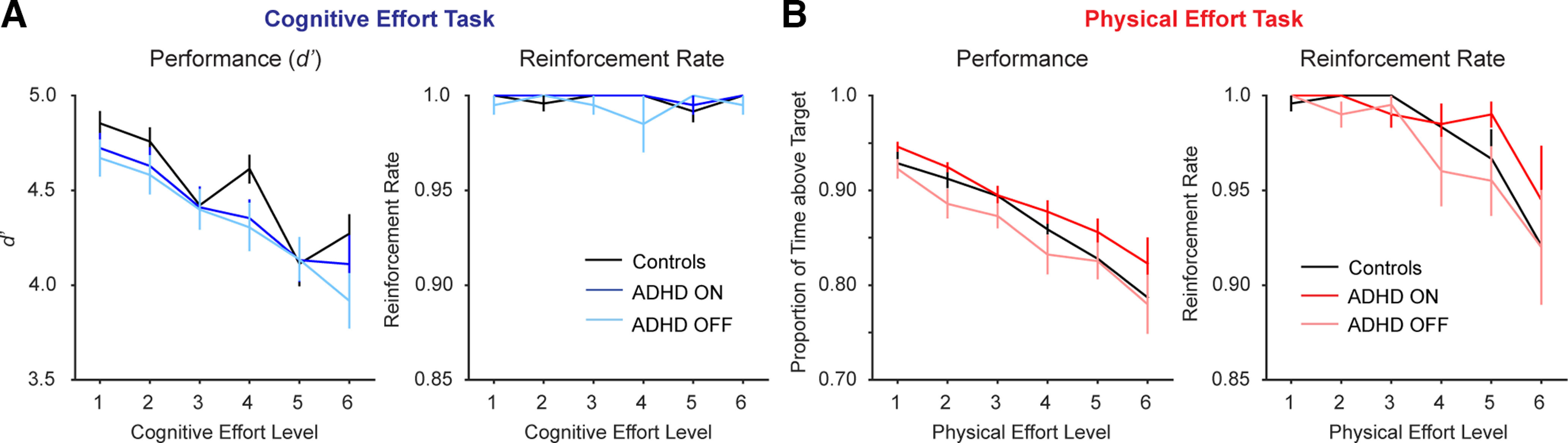
Data from the reinforcement phase. ***A***, In the cognitive effort task, there was a decrement in performance (*d’*) with increasing effort, but no significant group differences (left panel). Despite the decrement in performance, participants in both groups were able to perform each level of the task to the threshold required for reward (the reinforcement rate), (right panel). ***B***, In the physical effort task, there was also a decrement in performance (time above target) with increasing effort. However, task performance was greater in the ADHD group ON versus OFF medication (left panel). Unlike the cognitive effort task, the decrement in performance was mirrored by a decrement in reinforcement rate, although group differences were not significant (right panel).

First, we considered the capacity of the control and ADHD groups (both ON and OFF medication) to perform each level of the cognitive and physical effort tasks (Fig. 2).

#### Cognitive effort task

##### Task performance

We quantified cognitive performance as target detection sensitivity, *d’* [*Z*(Hits) – *Z*(False alarms)]. To determine the effect of Drug on *d’* in the ADHD group, we performed a within-subjects repeated-measures ANOVA on the factors of Drug (ON, OFF) and Effort (levels 1–6). As expected, there was a significant main effect of Effort, indicating an expected decrement in performance with increase in task load (*F*_(2.77,52.6)_ = 15.2, *p *<* *0.001). Importantly, however, neither the main effect of Drug, nor the Drug × Effort interaction, were significant (Drug, *F*_(1,19)_ = 0.48, *p = *0.50; Drug × Effort, *F*_(5,95)_ = 0.41, *p = *0.84).

We then compared the performance of controls against the ADHD groups ON and OFF medication using separate two-way ANOVAs with the between-subjects factor of Group (ADHD, controls), and within-subjects factor of Effort. Both ANOVAs revealed the same pattern of results – there was a significant main effect of Effort, which reiterated the decrement in performance with increasing load. However, there were no significant Group differences (Controls vs ADHD ON: Effort, *F*_(5,210)_ = 20.7, *p *<* *0.001; Group, *F*_(1,42)_ = 1.43, *p = *0.23; Group × Effort, *F*_(5,21)_ = 0.77, *p = *0.57; Controls vs ADHD OFF: Effort, *F*_(5,210)_ = 24.0, *p *<* *0.001; Group, *F*_(1,42)_ = 2.66, *p = *0.11; Group × Effort, *F*_(5,210)_ = 1.80, *p = *0.11).

##### Reinforcement rates

A complementary measure of performance is the capacity of individuals to perform each level to the threshold required for reward. For each level, we defined reinforcement rate as the proportion of trials for that level in which participants were able to successfully reach or exceed the reward threshold. To determine the effect of Drug (in ADHD participants) or Group (Control vs ADHD) on reinforcement rates, we performed the analogous ANOVAs as above. None of these analyses revealed any significant main effects or interactions (ADHD ON vs OFF: Drug, *F*_(1,19)_ = 1.73, *p = *0.20; Effort, *F*_(5,95)_ = 0.45, *p = *0.81; Drug × Effort, *F*_(5,95)_ = 0.83, *p = *0.53; Controls vs ADHD ON: Group, *F*_(1,42)_ = 0.72, *p *=* *.* *40; Effort, *F*_(5,210)_ = 2.10, *p = *0.067; Group × Effort, *F*_(5,210)_ = 0.28, *p = *0.93; Controls vs ADHD OFF: Group, *F*_(1,42)_ = 0.95, *p = *0.34; Effort, *F*_(5,210)_ = 0.34, *p = *0.89; Group × Effort, *F*_(5,210)_ = 1.31, *p = *0.26). Thus, participants in all groups were reinforced equally well across all effort levels.

In sum, these analyses revealed no significant differences in performance (in terms of *d’* or reinforcement rates) between the ADHD participants ON and OFF medication, or between the ADHD group and controls. This implies that any subsequent differences in the willingness of one group to exert higher levels of cognitive effort than another cannot be attributed to a differential capacity in task performance.

#### Physical effort task

##### Task performance

We performed the analogous set of analyses on the physical effort task. In this task, we operationalized performance as the proportion of the 10 s window in which individuals were able to sustain their force at or above the target effort level. When comparing the performance of ADHD participants ON and OFF medication, the main effect of Drug was significant, such that the ADHD group were able to sustain their force for longer when ON versus OFF medication (Drug, *F*_(1,19)_ = 7.25, *p = *0.014). The main effect of Effort was also significant, indicating that performance declined with increasing effort (Effort, *F*_(1.46,27.8)_ = 24.4, *p *<* *0.001). The two-way interaction was not significant (*F*_(2.58,49.0)_ = 0.65, *p = *0.57).

When comparing Controls against the ADHD group, the pattern of results was the same regardless of whether the ADHD participants were ON or OFF medication. In both cases, there was a significant main effect of Effort, but there were no statistically significant differences in task performance between the groups (Controls vs ADHD ON: Effort, *F*_(1.53,64.1)_ = 45.6, *p *< 0.001; Group, *F*_(1,42)_ = 1.30, *p = *0.26; Group × Effort, *F*_(1.53,64.1)_ = 0.71, *p = *0.46; Controls vs ADHD OFF: Effort, *F*_(1.85,77.9)_ = 49.8, *p *<* *0.001; Group, *F*_(1,42)_ = 0.52, *p = *0.48; Effort × Group, *F*_(1.85,77.9)_ = 0.55, *p = *0.56).

##### Reinforcement rate

When examining reinforcement rates in the ADHD group ON versus OFF medication, there was a significant main effect of Effort (*F*_(1.51,28.6)_ = 5.49, *p = *0.015), but neither the main effect of Drug nor the two-way interaction was significant (*F*_(1,19)_ = 1.77, *p = *0.20; Drug × Effort, *F*_(1.97,37.5)_ = 1.40, *p = *0.26). This same pattern was reiterated in the ANOVAs comparing controls to ADHD ON and OFF medication (Controls vs ADHD ON: Effort, *F*_(1.47,61.8)_ = 9.01, *p = *0.001; Group, *F*_(1,42)_ = 0.48, *p = *0.49; Effort × Group, *F*_(1.47,61.8)_ = 0.67, *p = *0.47; Controls vs ADHD OFF: Effort, *F*_(1.73,72.6)_ = 11.8, *p *<* *0.001; Group, *F*_(1,42)_ = 0.35, *p = *0.56; Effort × Group, *F*_(1.73,72.6)_ = 0.30, *p = *0.71).

Together, these analyses on the physical effort task indicate that individuals with ADHD were more capable of sustaining their force when ON versus OFF medication. However, this did not translate to a difference in the capacity of participants with ADHD to successfully obtain rewards. Notably, there were no differences between the control and ADHD groups in either task performance or reinforcement rate.

### Choice phase

#### Control data

Before considering the ADHD data, we first confirmed that the effort-based decisions in control participants replicated previous work. We analyzed choice data in controls with a generalized linear mixed effects model, which allowed us to assess all variables of interest on every trial. Specifically, we used a logistic transform to analyze Choice (baseline, offer) as the outcome variable, and entered Domain (cognitive, physical), Effort (levels 2–6), and Reward (2–10 credits) as fixed effects. Individual subjects were fit with random intercepts. Predictor variables were Z-scored, and we modeled the full range of fixed effects and interactions. Data were analyzed using the *fitglme* function in MATLAB with a logistic link function and Laplace fit method.

**Figure 3.**
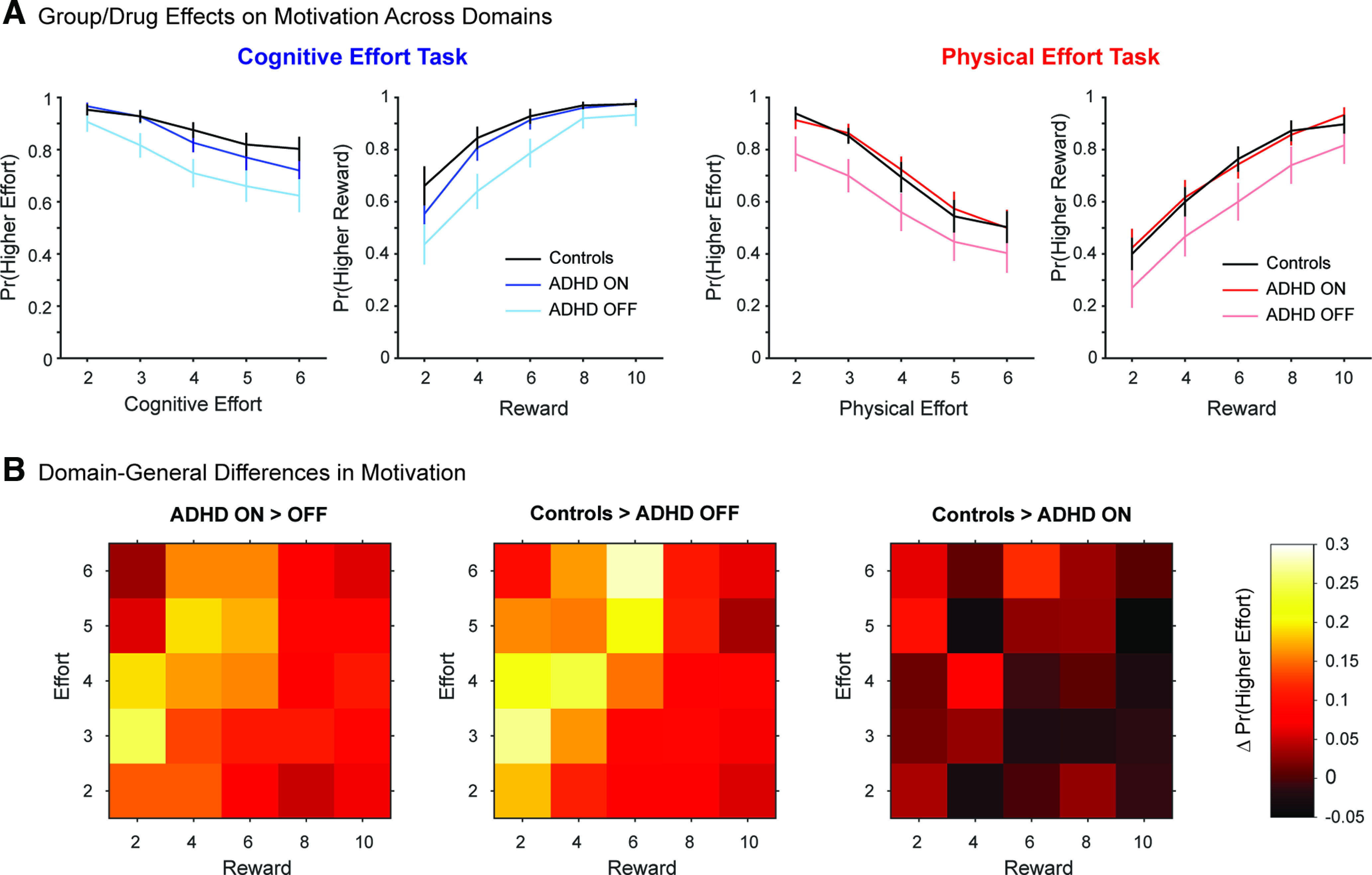
Overall differences in motivation between the control and ADHD groups, and the ADHD group ON and OFF medication. ***A***, The probability of choosing the more effortful option is shown as a function of increasing effort or reward. Choices in the cognitive task are shown on the left, and the physical task on the right. For each panel, separate plots compare choices for the control group (black) and the ADHD group ON (darker blue/red) and OFF (lighter blue/red) medication. ***B***, Choice difference between Group or Drug across the effort-reward space. The left panel shows the difference in probability of choosing the more effortful option in ADHD OFF medication from ADHD ON medication for each combination of effort and reward. The middle panel shows the analogous differences when subtracting ADHD OFF medication from controls, and the right panel when subtracting ADHD ON medication from controls.

The analysis of control data revealed a pattern of results similar to that described in previous studies (Chong et al., 2017; McGuigan et al., 2019; Atkins et al., 2020). All main effects were significant, and qualified by significant Domain × Effort (β = −0.42, *t =* −4.97, *p *<* *0.001) and Effort × Reward (β = 0.17, *t = *2.05, *p = *0.040) interactions, and a borderline Domain × Effort × Reward interaction (β = 0.15, *t = *1.80, *p = *0.072; Fig. 3*A*). The Domain × Effort interaction revealed that individuals were more likely to engage in cognitive versus physical effort at all but the lowest level of Effort (levels 3–6). The Effort × Reward interaction revealed that individuals were less inclined to exert higher levels of effort (i.e., effort discounting), but that this effect was greatest at the lowest levels of reward, and minimal at the highest levels of reward (i.e., higher rewards incentivized individuals to overcome all levels of effort). The borderline three-way interaction suggested that this effect was more pronounced in the physical domain, such that cognitive effort discounting was present across all rewards, but physical effort discounting only at lower rewards (two, four, six credits).

#### Effect of treatment (ADHD OFF vs ON)

Next, we examined the effect of stimulant medication on improving motivation in the ADHD group. We analyzed the ADHD data using a similar hierarchical generalized linear mixed effects model on the fixed effects of Domain, Effort and Reward, but now with the inclusion of Drug (OFF, ON) as an additional fixed effect. We again modeled the full range of fixed effects and interactions.

All simple effects were significant (Drug, β = 0.74, *t = *11.5, *p *<* *0.001; Domain, β = −0.80, *t =* −12.4, *p *<* *0.001; Effort, β = −1.23, *t =* −19.9, *p *<* *0.001; Reward, β = 1.69, *t = *26.5, *p *<* *0.001). Importantly, the effect of Drug was not qualified by any higher order interactions involving Domain (Drug × Domain × Effort, β = 0.064, *t = *1.06, *p = *0.29; Drug × Domain × Reward, β = −0.099, *t =* −1.62, *p = *0.10; Drug × Domain × Reward × Effort, β = 0.044, *t = *0.77, *p = *0.44). Rather, the effect of Drug was only involved in significant two-way and three-way interactions with Effort and Reward (Drug × Effort, β = −0.22, *t =* −3.61, *p *<* *0.001; Drug × Reward, β = 0.17, *t = *2.72, *p = *0.006; Drug × Effort × Reward, β = −0.18, *t =* −3.11, *p = *0.0018). This pattern of results indicates that Drug had a significant effect on choices, and in a similar manner across both the cognitive and physical domains.

The three-way Drug × Effort × Reward interaction indicated that the ADHD group were generally more motivated to exert effort when ON versus OFF at the lower effort levels (levels 2–4). However, at the higher effort levels (levels 5–6), the effects of Drug were more pronounced at intermediate levels of reward (four, six credits; Fig. 3*B*, left panel). The only other significant result was independent of Drug. Specifically, the Domain × Effort × Reward interaction was significant (β = 0.14, *t = *2.44, *p = *0.015), and simply reiterated the corresponding interaction in Controls, such that effort discounting was present at all reward levels in the cognitive domain, but only at the lower levels of reward in the physical domain. No other interactions were significant (all *p*-values > 0.07). Together, these results indicate that Drug had a significant effect on both Effort and Reward, but that these effects of Drug did not differentially affect one domain over another.

Recall that, in the training phase of this study, the ADHD group were less able to sustain their physical force when OFF versus ON medication. To ensure that the lower physical motivation in the ADHD group OFF versus ON medication was indeed because of the aversiveness of effort, rather than a decrement in the capacity to sustain a more effortful contraction, we performed a more complex GLM, with the inclusion of “Performance” of the more effortful option as an additional fixed effect. To do so, we first normalized performance for the cognitive and physical tasks separately (i.e., *d’* for the cognitive task, and time-above-target for the physical task). We then included Performance as an additional fixed effect to the reference model above. Importantly, however, this more complex model with Performance as an additional fixed effect led to a significantly poorer overall model fit (ΔAIC 1424, ΔBIC 1431 relative to the reference model). Furthermore, this more complex model revealed the same pattern of significant results as in the reference analysis. Specifically, the effect of Drug was not qualified by any higher order interactions involving Domain (Drug × Domain × Effort, β = 0.08, *t = *1.32, *p = *0.19; Drug × Domain × Reward, β = −0.11, *t =* −1.77, *p = *0.08; Drug × Domain × Reward × Effort, β = 0.05, *t = *0.77, *p = *0.44). Again, Drug was only involved in significant two-way and three-way interactions with Effort and Reward (Drug × Effort, β = −0.23, *t =* −3.74, *p* < 0.001; Drug × Reward, β = 0.16, *t = *2.61, *p = *0.009; Drug × Effort × Reward, β = −0.19, *t =* −3.24, *p = *0.001).

For completion, we performed a complementary analysis on Reinforcement Rates to ensure that effort aversion was not driven by the capacity to perform each level to the required reward threshold. We included the normalized Reinforcement Rates of the more effortful option as an additional fixed effect to the reference model. Again, however, this more complex model revealed a poorer overall fit (ΔAIC 565, ΔBIC 572 relative to the reference model), and the pattern of the results remained unchanged. Again, the effect of Drug was not qualified by any higher order interactions involving Domain (Drug × Domain × Effort, β = 0.05, *t = *0.81, *p = *0.42; Drug × Domain × Reward, β = −0.10, *t =* −1.69, *p = *0.10; Drug × Domain × Reward × Effort, β = 0.04, *t = *0.65, *p = *0.51). As in the reference analysis, the only significant interactions involving Drug were two-way and three-way interactions with Effort and Reward (Drug × Effort, β = −0.22, *t =* −3.56, *p* < 0.001; Drug × Reward, β = 0.16, *t = *2.62, *p = *0.009; Drug × Effort × Reward, β = −0.18, *t =* −3.07, *p = *0.002).

Finally, to test for potential session order effects (ON-OFF vs OFF-ON), we modified the reference model by including fixed effects of Order, and an Order × Drug interaction term. Importantly, this model did not substantially improve the model fit relative to the reference model (ΔAIC 230, ΔBIC 243). Furthermore, the Order × Drug interaction term was not significant (β = 0.54, *t = *0.91, *p = *0.36), and the inclusion of these additional terms did not alter the pattern of the remaining effects and interactions. Specifically, the effect of Drug was not qualified by any higher order interactions involving Domain (Drug × Domain × Effort, β = 0.06, *t = *1.02, *p = *0.31; Drug × Domain × Reward, β = −0.10, *t =* −1.58, *p = *0.12; Drug × Domain × Reward × Effort, β = 0.05, *t = *0.80, *p = *0.42), and the only significant interactions with Drug were two-way and three-way interactions with Effort and Reward (Drug × Effort, β = −0.22, *t =* −3.63, *p* < 0.001; Drug × Reward, β = 0.17, *t = *2.74, *p = *0.006; Drug × Effort × Reward, β = −0.17, *t =* −3.04, *p = *0.002).

In summary, these results indicate that the ADHD group were more motivated to invest both cognitive and physical effort when ON medication relative to OFF, and that this result could not be explained by their ability to perform these tasks, their capacity to complete them to the required threshold, or the order in which they were tested.

#### Effect of diagnosis (Controls vs ADHD OFF)

We then considered the behavior of the ADHD group OFF medication relative to healthy controls (Fig. 3). We performed a similar GLM, replacing the within-subjects factor of Drug with a between-subjects factor of Group (ADHD, controls). All other aspects of this analysis were identical to the preceding model. The main effects of Domain, Effort and Reward were significant (Group, β = −0.65, *t* = −1.54, *p = *0.12; Domain, β = −0.88, *t* = −15.6, *p *<* *0.001; Effort, β = −1.21, *t* = −21.0, *p *<* *0.001; Reward, β = 1.72, *t* = 28.5, *p *<* *0.001). Notably, Group did have a differential effect on motivation across the two domains. A significant Group × Domain × Effort interaction (β = 0.14, *t* = 2.59, *p = *0.01) indicated that controls were more motivated than ADHD OFF medication at the higher levels of Cognitive effort (levels 3–6), and lower levels of Physical effort (levels 2–3; Fig. 3*A*).

The only other significant results were independent of Group, and involved a series of two-way and three-way interactions including Domain, Effort and Reward, which reiterated the same pattern of cognitive and physical effort discounting as described in the preceding analyses (Domain × Effort, β = −0.29, *t =* −5.31, *p *<* *0.001; Effort × Reward, β = 0.22, *t = *3.97, *p = *0.0001; Domain × Effort × Reward, β = 0.13, *t = *2.41, *p = *0.016; all other interactions, *p *>* *0.15).

As in the ADHD ON versus OFF data, we ensured that this pattern of results was not driven by a reduced capacity of individuals to perform the higher levels of effort. In two separate GLMs, we again included Performance and Reinforcement Rates as additional fixed effects. Both GLMs provided better fits to the reference model comparing Control performance to ADHD OFF medication (Performance, ΔAIC = −111, ΔBIC = −104; Reinforcement Rates, Δ AIC = −42, Δ BIC −36). Importantly, however, the pattern of significant main effects and interactions was the same across both GLMs and the reference model. Thus, the results above were not modulated by the capacity of individuals to be able to perform the higher levels of cognitive or physical effort.

#### Efficacy of treatment (Controls vs ADHD ON)

Finally, we considered the efficacy of drug treatment by comparing the ADHD group ON medication to the control group. We implemented the same model as reported for ADHD OFF versus controls, but this time using data from the ADHD ON group. Again, the main effects of Domain, Effort and Reward were significant (Group, β = −0.004, *t =* −0.01, *p = *0.99; Domain, β = −0.90, *t =* −12.8, *p *<* *0.001; Effort, β = −1.38, *t =* −20.0, *p *<* *.* *001; Reward, β = 1.82, *t = *25.8, *p *<* *0.001). Notably, these effects were involved in higher-order two-way and three-way interactions that indicated a significant differential effect of Group on choices in the cognitive and physical Domains (Group × Domain × Effort, β = 0.21, *t = *3.07, *p = *0.002; Group × Domain × Reward, β = −0.17, *t =* −2.57, *p = *0.01; Fig. 3*A*). These interactions showed that controls and ADHD ON medication did not differ in physical motivation; rather, any group differences were in cognitive motivation. Specifically, the Group × Domain × Effort interaction was driven by controls being more motivated than the ADHD group ON medication at higher levels of cognitive effort (levels 4–6). The Group × Domain × Reward interaction indicated higher cognitive motivation in controls than ADHD ON at the lowest level of reward (two credits).

The other significant interactions involving Group were independent of Domain. There were significant Group × Effort (β = −0.14, *t =* −2.00, *p = *0.045) and Group × Effort × Reward (β = −0.14, *t =* −2.22, *p = *0.03; Fig. 3*B*, right plot) interactions. These indicated that controls were more motivated than the ADHD group only at the highest levels of effort at the intermediate level of Reward (6 credits). Finally, there were significant Domain × Effort (β = −0.23, *t =* −3.42, *p *<* *0.001) and Domain × Effort × Reward (β = 0.16, *t = *2.57, *p = *0.01) interactions, which simply reiterated the domain-specific patterns of effort discounting described in the preceding analyses.

Control analyses again revealed that including Performance or Reinforcement Rates did not improve overall model fits relative to the preceding reference model (Performance, ΔAIC 469, ΔBIC 477; Reinforcement Rates, ΔAIC 160, ΔBIC 167). These control models also revealed the same pattern of results as the reference model.

#### No correlations between willingness to invest effort, and apathy or ADHD severity

As supplementary analyses, we probed for potential relationships between the willingness to engage in effortful behavior, and measures of trait apathy. Specifically, we used a Spearman’s ρ to correlate acceptance rates in the ADHD group (averaged across both drug sessions), against scores on each subscale of the DAS. We analyzed this relationship for acceptance rates in the cognitive and physical tasks separately, as well as when averaged across both tasks. However, none of these correlations were significant (cognitive task, all |ρ| < 0.38, all *p* > 0.10; physical task, |ρ| < 0.14, *p* > 0.55; both tasks, |ρ| < 0.27, *p* > 0.25). We also tested for a correlation between acceptance rates, and each subscale of the CAARS and WURS. Again, however, none of the correlations were significant (cognitive task, |ρ| < 0.32, *p* > 0.17; physical task, |ρ| < 0.33, *p* > 0.15; both tasks, |ρ| < 0.31, *p* > 0.19).

## Discussion

In this study, we asked whether individuals with ADHD demonstrated altered effort sensitivity relative to controls, and, if so, how these changes can be ameliorated by amphetamines. Our data revealed three main findings. First, ADHD participants OFF medication were less motivated than controls to invest effort in both the cognitive and physical domains. Second, amphetamines increased motivation to a similar degree in both domains. Finally, the net effect of amphetamine treatment was to restore motivation to levels very similar to controls, with only minor group differences persisting at the highest level of cognitive effort. Overall, our data provide clear evidence that effort sensitivity is impaired in ADHD, and indicate the efficacy of amphetamine-based medications in treating this impairment.

An aversion to cognitive effort is thought to play a key role in the cognitive inefficiencies in ADHD (Sergeant, 2005). Indeed, several theoretical models have proposed that the attentional and executive deficits in ADHD are secondary to a motivational impairment (Nigg, 2005; Tripp and Wickens, 2009; Luman et al., 2010), and specifically a reduction in the willingness to expend cognitive energy (Hsu et al., 2017). Until now, however, no study has explicitly tested the proposition that ADHD is associated with a deficit in cognitive motivation. Here, we provide the first direct evidence that the willingness to engage in cognitively effortful activity is reduced in individuals with ADHD when they are OFF medication relative to controls. An outstanding question for future work is whether this aversion to effort is causally related, either wholly or in part, to the apparent cognitive inefficiencies that are typical of the cognitive profile in ADHD (Sergeant, 2005).

Our data also showed that individuals with ADHD had a clear aversion to physical effort when tested OFF medication relative to controls. At first glance, this may appear to contrast with the only other studies that have examined physical effort in ADHD, none of which have shown strong support for effort aversion (Winter et al., 2019; Mies et al., 2018; Addicott et al., 2019). However, in one study, there was no formal comparison between ADHD participants OFF medication and controls (Addicott et al., 2019); and, in the remaining two, the medication status of child and adolescent participants were difficult to control (Winter et al., 2019; Mies et al., 2018). Specifically, in the latter two studies, participants were tested at most 24 h after their last dose, which leaves open the possibility that residual medication effects may have obscured any fundamental group differences in physical motivation. Indeed, our data indicate that, when ADHD participants are tested ON medication, amphetamines restored their physical motivation to levels indistinguishable from controls.

Prevailing frameworks propose that a motivational deficit is one of the key characteristics of ADHD (American Psychiatric Association, 2022). Some have proposed that the impaired motivation in ADHD is because of dysfunction of the mesocorticolimbic pathways, in which dopamine and noradrenaline play important roles. For example, lower trait motivation in ADHD has been associated with lower availability of the dopamine D2/D3 receptor and dopamine transporter (Volkow et al., 2011). The mesocorticolimbic pathway has been heavily implicated in effort allocation (Walton et al., 2006; Hamid et al., 2016; Lopez-Gamundi et al., 2021; Suzuki et al., 2021), and striatal dysfunction leads to a heightened aversion to both cognitive and physical effort (Wolf et al., 2014; Chong et al., 2015; McGuigan et al., 2019; Atkins et al., 2020). Our data provide evidence to support the prediction that ADHD is associated with greater effort sensitivity, and that these motivational changes are comparable across both the cognitive and the physical domains.

In our study, we operationalized motivation as a reduced willingness to engage in cognitively or physically effortful behavior (Pessiglione et al., 2018; Chong, 2018; Husain and Roiser, 2018). Notably, differences in motivation between the control and ADHD groups persisted even after accounting for the capacity of individuals to perform the tasks themselves. Indeed, in the reinforcement phase (when participants had no choice about which levels to perform), the capacity of both groups to perform either the cognitive or physical effort task was similar. However, in the choice phase (when participants voluntarily decided which effort levels they preferred), the ADHD group OFF medication demonstrated a clear aversion to effort relative to controls. In other conditions, the aversiveness of effort has been linked to apathetic traits (McGuigan et al., 2019; Atkins et al., 2020; Le Bouc et al., 2023). However, this finding has not been ubiquitous, due partly to heterogeneous approaches in how both effort and trait apathy have been assessed (Hartmann-Riemer et al., 2018; McLauchlan et al., 2019). Thus, although our analyses did not reveal any correlations between effort-based decisions and trait apathy, it remains for future studies to clarify this relationship in ADHD.

Our data also showed that amphetamines had a domain-general effect on improving motivation. The only other study to have examined the effect of stimulants on effort-based decisions showed that the motivation to invest physical effort was higher in ADHD participants on methylphenidate versus placebo (Addicott et al., 2019). Importantly, however, this study used an effort manipulation in which the more effortful levels (more button presses) also took longer to complete. It is therefore difficult to ascribe any effect of drug specifically to an aversion to effort versus an aversion to delay. This is an important consideration given that greater delay discounting is a consistent finding in ADHD (Sonuga-Barke et al., 2008; Jackson and MacKillop, 2016; Patros et al., 2016). In contrast, the temporal demands of our task were matched across all effort levels, which allows us to exclude the possibility that our findings on effort discounting were confounded by delay.

Overall, our data provide causal support for the importance of catecholamines in overcoming both cognitive and physical effort costs in pursuit of a goal. Dopamine and noradrenaline both play critical roles in facilitating effortful behavior. For example, a large body of work indicates that dopamine incentivizes and invigorates approach behavior for reward (De Jong et al., 2015; Salamone et al., 2016; Soares-Cunha et al., 2016; Chong, 2018; Gallo et al., 2018; Michely et al., 2020; Salamone et al., 2022). Traditionally, noradrenaline has been known to play an important role in arousal, but recent data indicate that it also specifically encodes the amount of effort required to accomplish a goal (Varazzani et al., 2015; Jahn et al., 2018; Borderies et al., 2020; Poe et al., 2020). Many studies have focused on the causal role of these catecholamines in motivating physical effort in humans (Wardle et al., 2011; Soder et al., 2021; for review, see Salamone et al., 2016; Walton and Bouret, 2019). The role of catecholamines in cognitive effort has been relatively less explored, but emerging data indicate a comparable effect (Cocker et al., 2012; McGuigan et al., 2019; Westbrook et al., 2020; Bogdanov et al., 2022). Importantly, no single study has directly compared the effects of catecholaminergic stimulation within participants across both domains. Our data therefore provide important support for the idea that catecholamines are important in supporting motivation across multiple domains of behavior. More broadly, they add to growing evidence suggesting that catecholaminergic drugs may be efficacious in treating disorders of motivation, such as apathy, in appropriate clinical groups (Chong and Husain, 2016; Mintzer et al., 2021; David et al., 2022).

Although much of the focus in ADHD has been on dopamine and noradrenaline dysfunction, it is important to note that serotonin is another monoamine neurotransmitter that is important in goal-directed behavior. In our study, ADHD participants were managed with either dexamfetamine, or its prodrug, lisdexamfetamine (Castells et al., 2018). The primary effect of dexamfetamine is to promote dopamine release and inhibit its reuptake, with secondary effects on noradrenaline (Kuczenski et al., 1995; Hedou et al., 2000; Shi et al., 2000). Amphetamines also augment the serotonergic system, although their effects on serotonin are thought to be less pronounced than on dopamine and noradrenaline transmission (Gatley et al., 1996). The role of serotonin in effort allocation is less well established, but recent data suggest that serotonin may also motivate effortful behavior by reducing the perceived cost of effort (Meyniel et al., 2016). Given the complex interactions between the dopaminergic and serotonergic systems, it remains for future work to clarify the relative contribution of serotonin to the effects we observed here (Dremencov et al., 2009; Schilström et al., 2011; Cools, 2015; Fischer et al., 2015).

In summary, we provide evidence for effort aversion as an important mechanism for the reduced motivation in ADHD individuals, and show that treatment with amphetamine-based drugs is capable of restoring motivation to levels comparable to those in healthy individuals. Our data indicate that the heightened effort aversion is experienced across multiple domains of effort, and is independent of the capacity of the individual to actually perform the task at hand. This provides further confirmatory evidence that the motivational deficits in ADHD are not secondary to dysfunction of particular cognitive domains; rather, it is likely to represent a primary dysfunction in these individuals. More broadly, our data add to accumulating evidence for the causal role of catecholamines in increasing motivated behavior, and provide further evidence for the in-principle efficacy of such drugs to improve apathy in appropriate clinical populations.
